# Radiomic Features Extracted from Magnetic Resonance Imaging to Identify Arteriopathy in a Humanized Mouse Model of Sickle Cell Anemia and Sickle Cell Trait

**DOI:** 10.1007/s10439-026-04034-8

**Published:** 2026-02-23

**Authors:** Liana Hatoum, Viviana Benfante, Hannah Song Lee, Edward A. Botchwey, Albert Comelli, Manu O. Platt

**Affiliations:** 1 Interdisciplinary Bioengineering Graduate Program, Georgia Institute of Technology, Atlanta, GA USA; 2 Wallace H. Coulter Department of Biomedical Engineering, Georgia Institute of Technology and Emory University, Atlanta, GA USA; 3 Ri.MED Foundation, Via Bandiera, 11, 90133 Palermo, Italy; 4 Department of Health Promotion, Mother and Child Care, Internal Medicine and Medical Specialties, Molecular and Clinical Medicine, University of Palermo, Palermo, Italy; 5 NIH Center for Biomedical Engineering Technology Acceleration, Bethesda, MD USA; 6 National Institute of Biomedical Imaging and Bioengineering, National Institutes of Health, Bethesda, MD USA; 7 National Institutes of Health, 9000 Rockville Pike, Bldg 35A, Room GD-937, Bethesda, MD 20892 USA

**Keywords:** Sickle cell disease, Radiomics, Magnetic resonance angiography, Translational

## Abstract

**Purpose:**

Sickle cell disease (SCD) is a hereditary blood disorder that increases stroke risk in children. Previously, we showed that SCD caused accelerated arteriopathy in a sickle cell transgenic mouse model compared to sickle cell trait controls. We sought to determine whether radiomics analysis of label-free carotid artery magnetic resonance angiography (MRA) can differentiate between SCD mice (SS) from heterozygous sickle cell trait control mice (AS). Radiomics analysis of MRI data was used to extract quantitative imaging features, then tested for discrimination between SS and AS mice.

**Methods:**

MRA scans of Townes sickle cell transgenic mice at one and three months of age were completed. 112 radiomic features extracted from segmented carotid artery images using PyRadiomics software were used to develop models predictive of SCD genotypes.

**Results:**

At one month of age, four radiomics features yielded accuracy of 74%. At three months, a single feature achieved 76% accuracy. Analysis of MRA-derived arterial morphology confirmed that incorrectly identified mice carotid arteries resembled the incorrectly predicted genotype: larger luminal areas for AS and smaller luminal areas for SS, reflecting how biological variability of SCD impacted radiomic feature predictions.

**Conclusion:**

This study demonstrates feasibility of radiomics in discriminating arterial features between SCD and control mice, and supports radiomics as a non-invasive imaging analytical approach to characterize arterial remodeling in preclinical SCD models, motivating future translational studies linking imaging features to vascular pathology.

## Introduction

Sickle cell disease (SCD) is an inherited hemoglobinopathy affecting approximately 100,000 people in the United States, with significantly higher prevalence in sub-Saharan Africa, India, and the Middle East [1, 2]. SCD causes chronic hemolysis of red blood cells and vaso-occlusion, causing multi-organ damage, frequent pain episodes, and a shortened life expectancy [3–5]. Among its most devastating complications are cerebrovascular events—both overt ischemic and hemorrhagic stroke as well as clinically silent stroke—which collectively drive significant morbidity and mortality [6]. Despite notable advances in screening with transcranial Doppler (TCD) and treatment via blood transfusions and hydroxyurea therapy, stroke risk remains substantial [7], underscoring the need for biomarkers to identify high-risk patients before irreversible vascular damage occurs. Radiomics has emerged as a promising approach to extract high-dimensional, quantitative information from medical images [8–10]. Originally developed within oncology, radiomics transforms imaging data into interpretable features capturing tissue texture, shape, and intensity distributions [11]. By leveraging machine learning, these features can be integrated into predictive models for diagnosis, risk stratification, and outcome prediction [12, 13]. Radiomics has proven valuable in cardiovascular imaging, where it identified high-risk atherosclerotic plaques [14–16] and detected subtleties that are often imperceptible to the human eye [17]. These studies demonstrate that quantitative image features add diagnostic power beyond conventional imaging alone.

For SCD, magnetic resonance imaging and angiography (MRI/MRA) have become indispensable for evaluating cerebrovascular health. MRI/MRA offers superior soft-tissue contrast without radiation exposure [18, 19]. These advantages are especially critical for patients requiring periodic assessment, such as children and young adults with SCD [6]. MRI-based measurements can quantify large-artery pathology such as carotid expansion, elastic lamina disruption, and abnormal tortuosity [19, 20]. However, many individuals with SCD develop impaired kidney function over time [21], limiting the feasibility of gadolinium-enhanced MRA. Non-contrast techniques such as time-of-flight (TOF) MRA provide an attractive alternative but can be more challenging in larger mammals, including humans, due to flow-related signal loss and reduced sensitivity to subtle vascular abnormalities. A radiomics approach may help overcome these limitations by extracting quantitative information from non-contrast MRA that is not readily appreciable by visual assessment alone.

The primary objectives of this study are: [1] to investigate if MR-based radiomic features can discriminate between homozygous sickle cell disease (SS), and the heterozygous condition, equivalent of sickle cell trait in humans (AS), with unbiased approaches, and [2] to explore the feasibility of using radiomics to improve non-invasive risk stratification strategies in SCD. By applying rigorous feature extraction, selection, and machine learning protocols [11, 20], we establish the groundwork for future clinical studies that implement personalized radiomics-based risk models in patient-clinician decisions and SCD management.

## Materials and Methods

### Animals

We used Townes sickle cell mice (B6; 129-Hbatm1(HBA) Tow Hbbtm2 (HBG1, HBB*) Tow/Hbbtm3 (HBG1, HBB) Tow/J) at Georgia Institute of Technology, originally purchased from The Jackson Laboratory. Genotypes are homozygous for *β*-globin S mutation (SS) or, littermate heterozygous (AS; equivalent to sickle cell trait in humans). Animals were kept in climate-controlled rooms on a 12-h light-dark cycle and allowed access to food and water ad libitum. Mice were phenotyped for sickle (HbS) and normal human hemoglobin (HbA) by native hemoglobin polyacrylamide gel electrophoresis using peripheral capillary blood obtained via paw puncture with an automated safety lancet. Both male and female SS and AS mice were used and summarized in Table 1. In approximate human developmental terms, 4-week-old mice correspond to late childhood/early adolescence, whereas 12-week-old mice correspond to young adulthood [22]. Animal handling and experimentation were conducted ethically and by approved Institutional Animal Care and Use Committee (IACUC) protocols at Georgia Institute of Technology.

**Table 1:** Distribution of mice by genotype, sex, and imaging age

Genotype	Sex	1 Month (*n*)	3 Months (n)	1 and 3 Months (n)
SS	Male	18	16	7
SS	Female	20	15	7
Total SS		**38**	**31**	**14**
AS	Male	11	14	5
AS	Female	17	19	6
Total AS		**28**	**33**	**11**
Overall Total		**66**	**64**	**25**

Bold values indicate totals for each subgroup and overall totals

The numbers for 1 and 3 months are included in the separate tallies as well

### Magnetic Resonance Angiography

All in vivo magnetic resonance angiography imaging was done with a 7 T MRI system (PharmaScan, BRUKER) with a Doty Scientific radiofrequency coil (inner diameter = 25 mm) and without the use of any contrast agents. All images were acquired using the same scanner at Georgia Institute of Technology. Mice were anesthetized by isoflurane inhalation delivered in 100% oxygen and maintained at 1–2%. During imaging, mice were placed in a prone position and covered with a circulating warm-water heating pad. Respiratory rate was continuously monitored via a small pneumatic pillow sensor secured on the animal’s abdomen (PC-SAM software by SA Instruments). MRA was performed with 3-dimensional time of flight (TOF) sequence of a repetition time of 14 ms, echo time of 2.6 ms, a flip angle of 20°, slice thickness of 20 mm, field of view of 20 × 20 × 20 mm with acquired matrix size of 256 × 256 × 128  mm, with no zero-filling or interpolation applied. The MRA acquisition time was approximately 13 minutes. Angiograms were obtained by generating maximal intensity projections (MIP) of the raw data.

### Image Processing

The medical image processing software, *Mimics* (Materialise, Leuven, Belgium) was used to segment and reconstruct common carotid arteries. A minimum threshold was selected visually for each case, which produced the largest vessel diameter while minimizing background noise and surface irregularities in the segmented carotid artery. After a threshold was chosen, a region regrowing tool was used to automatically segment the carotid and cerebral arteries. All vessels were removed except the common carotid arteries which were then isolated from their origin at the ascending aorta to the carotid bifurcation. Afterwards, common carotids were manually refined from background noise and subsequently smoothed using the Smoothing tool in *Mimics*. Vessel centerlines were generated using the semi-automatic centerline extraction tool. Cross-sectional area measurements were computed along the centerline over the full length of each artery at 0.15 mm intervals. The MRA scans and segmentation and reconstruction of carotid arteries were converted from the DICOM format to the 3D NIfTI (Neuroimaging Informatics Technology Initiative) volumetric format.

### Radiomics Analysis

A total of 66 MRI scans of carotid arteries were collected from one-month-old mice (*n* = 28 AS; *n* = 38 SS) and 64 MRI scans were collected from three-month-old mice (*n* = 33 AS; *n* = 31 SS). 25 mice were scanned at both 1 month and 3 months of age. After image acquisition, region of interest delineation and 3D shape reconstruction of carotid arteries, a radiomics analysis workflow was applied. The applied workflow consists of the following phases:

i) Extraction of radiomics features;
ii) Selection of radiomic features;
iii) K-fold strategy to split data into training and validation sets;
iv) Predictive model training and validation phase based on discriminant analysis.

Using PyRadiomics (3.0) software, radiomic features were extracted [23] compliant with the Biomarker Imaging Standardization Initiative (IBSI) [24], an independent international collaborative study aimed at standardizing radiomic features for segmented carotid artery lumens, which typically exhibit high-throughput quantitative image analysis [23]. Based on each segmented carotid artery’s volume, 112 radiomic features were automatically obtained. No filter was applied. We kept all the standard parameters except: ‘binWidth: 0.8’ to size the bins when making a histogram and for discretization of the image gray level and ‘normalize: True’ to enable normalization of the image before resampling. The extracted features include first order texture features (which provide information related to the distribution of the gray level within the ROI, without taking into account the spatial relationships between the voxels), second order (which take into account the spatial relationships between the voxels), and third order (which evaluate the spatial relationship between three or more voxels).

Based on the histogram analysis of gray levels (e.g., mean, variance, skewness, kurtosis, and percentiles), texture parameters were determined. To calculate the co-occurrence matrix, five measurements were taken (i.e., contrast, correlation, sum of squares, inverse difference moment [IDM], mean sum, variance sum, entropy sum, variance difference, entropy difference). To calculate the run length matrix, four directions were taken into account (such as sequence length nonuniformity [RLN], normalized gray level nonuniformity [GLNN], length run emphasis [LRE], and short run emphasis [SRE]). Radiomics parameters are described in detail in the PyRadiomics online documentation [23].

Feature selection was carried out using a hybrid statistical method independent from the operator, while the predictive model was developed using linear discriminant analysis. As a means of identifying the most discriminating features and decreasing redundancy between radiomic features with a high correlation, an innovative sequential mixed descriptive-inferential approach was used [11]. The method proposed here is based on the use of the point biserial correlation (*r*_pb_) index [25–27] a statistic especially adopted in psychometrics and social sciences. It appears very suitable to quantify the strength of the link between a feature and the classification response. In combination, logistic regression is adopted step-wise, with a minimum *p*-value convergence criterion. In the methodology presented here, there is no pre-filtering based on *r*_pb_ index, but only a pre-ranking, then the selection is made by the significance of the logistic regression model fitting. Such a specific procedure provides a new solution to this problem. Mice were classified into genotypes: homozygous for *β*-globin S mutation (SS) or, littermate heterozygous (AS). From a statistical viewpoint, this classification defines a column array Y (*n* x1), of a dichotomous variable usually called the gold standard. The extracted features obtained by the PyRadiomics software are continuous and categorical variables. The dataset is so composed, in addition to the column array Y, of an X array with n rows (mice, cases) and m columns (features). For statistical analysis, one problem is that the number of cases can be lower than the number of variables, *n* ≪ *m*. Besides, some columns of the X array can be characterized by very odd distributions. For this reason, any automated approach (e.g., support vector machine), trying to deal with the analysis of such datasets, without any exploratory preprocessing, can be very ineffective. Consequently, to identify the key features and to improve predictive modeling, a hybrid descriptive-inferential sequential approach is here proposed and was applied.

The adopted algorithm consists of the following steps:

- Step 1: For each column of X calculate the *r*_pb_ index with the column array Y;
- Step 2: Rearrange the columns of X by sorting them in descending order of absolute value of r_pb_;
- Step 3: Start a WHILE-DO cycle: by adding one column at time from the rearranged X matrix, perform a multiple logistic regression analysis. Consider the *p*-value of the Pearson’s chi square goodness of fit test. The smaller the *p*-value the better the logistic regression model interprets the variability of the response Y. Compare the *p*-value of the current iteration with the *p*-value of the previous iteration and quit the cycle when it has not decreased. The final model includes all regressors added in the previous cycle.

Consequently, the most discriminating features were identified.

Using linear discriminant analysis (predictive model), a predictive model was developed as in [8]. We trained linear discriminant analysis with the most discriminating features to evaluate differences between carotid arteries in sickle cell anemia (SS) mice and heterozygous sickle cell trait (AS) mice. As a gold standard for developing the training input for the classifier, an expert operator classified the carotid arteries in SS and AS mice. Using the k-fold strategy, the data was divided into training and validation sets. By doing so, studies were divided into k-folds. An individual fold was used as the validation set, while the remaining folds were combined to form the training set. In order to validate the model, each fold was used as the validation set and the remaining sets as the training sets. Using a trial-and-error strategy, we determined *k* = 5 empirically (*k* range: 5–15, step size: 5). Once the training task was completed, discriminant analysis was able to classify the most discriminatory features extracted from the carotid arteries of new mice into mice with sickle cell anemia (SS) and heterozygous with sickle cell trait (AS).

## Results

### Radiomics Analysis of One-month-old Mice Identified Four Features to Distinguish Between SS and AS Phenotypes

A total of 112 radiomic features were extracted from each volume of segmented common carotid arteries from 1-month-old to 3-month-old mouse cases, respectively, using PyRadiomics software. Based on the selection process, the following group of features significantly distinguished the two groups of one-month-old mice (Table 2):

**Table 2:** Discriminant radiomics features obtained from the radiomics analysis on one-month-old mouse cases

Discriminant radiomics features	*P*-value (< 0.05)
Original glcm Imc1	0.0420
Original first-order Minimum	0.0411
Original shape MinorAxisLength	0.0126
Original shape MajorAxisLength	0.0025

*Informational Measure of Correlation* (IMC) 1 assesses the correlation between the probability distributions of i and j (quantifying the complexity of the texture), using mutual information P(i, j) on Gray Level Co-occurrence Matrix (glcm).

First order minimum assesses.

$$minimum \, = \, \min \, \left( X \right)$$

X is a set of voxels included in the ROI

MinorAxisLength is the feature that yields the second-largest axis length of the ROI-enclosing ellipsoid and is calculated using the largest principal component λminor.

$$minor \, axis \, = \, 4\sqrt {\lambda minor}$$

MajorAxisLength is the feature that yields the largest axis length of the ROI-enclosing ellipsoid and is calculated using the largest principal component λmajor.

$$major \, axis \, = \, 4\sqrt {\lambda major}$$

The principal component analysis was performed using the physical coordinates of the voxel centers defining the ROI.

The “Informative Correlation Measure” (Imc1) feature represents a measure of texture complexity that could imply highly variable signal intensity across voxels of interest. The min(x) is simply the lowest signal intensity of a set of voxels within the ROI. The majoraxislength and the minoraxislength are the largest and smallest length axes, respectively, of the ROI-enclosing ellipsoid.

**Fig. 1:**
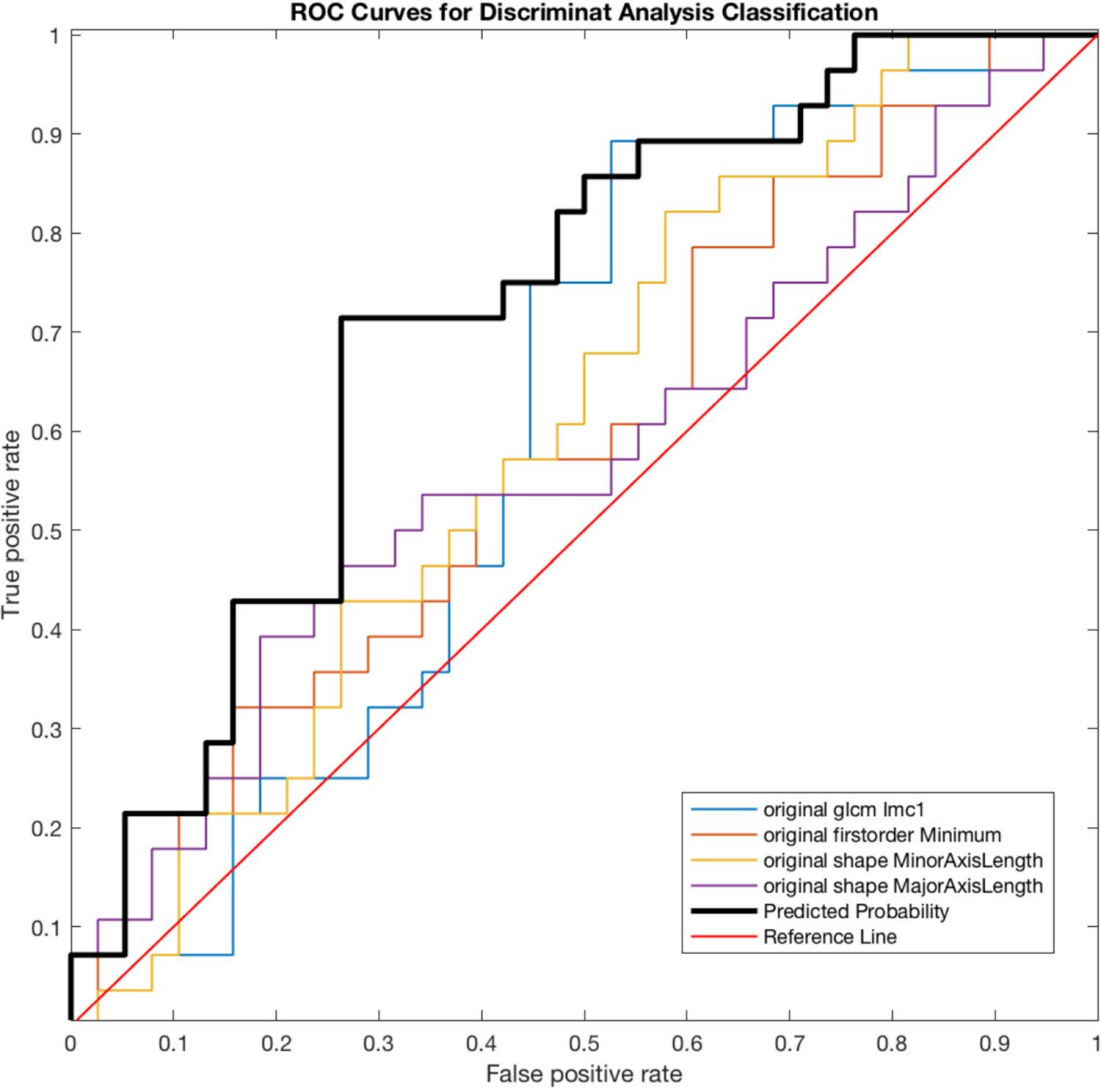
ROC curves for combination of the four most discriminated radiomic features in mice at 1 month of age (original glcm Imc1, First Order Minimum, shape MinorAxisLength, shape MajorAxisLength). The combination of these features showed statistically significant discrimination between SS and AS, with an AUROC of 0.72; 95% CI between 0.60 and 0.84 with *p*-value of 0.032

The combination of these features presents high diagnostic performance in discrimination between SS and AS, with an AUROC of 0.72; 95% CI between 0.60 and 0.84 with p-value of 0.032, sensitivity of 68.03%; 95% CI between 55.45 and 78.92, specificity of 82.16%; 95% CI between 71.58 and 89.57, an accuracy of 74.22%; 95% CI between 62.11 and 84.06 and a Positive Predictive Value % (PPV) of 84.31%; 95% CI between 73.97 and 91.26. Figure 1 shows the ROC curve for the combination of the four most discriminated radiomic features in mice at 1 month of age (Fig. 1).

### One Radiomic Feature is Sufficient to Distinguish Between 3-Month-Old SS and AS Phenotypes

Using three-month mouse MRA scans and radiomic feature extraction process described above, only one first-order texture feature, original shape MeshVolume, was sufficient to significantly discriminate between AS and SS mice (Table 3). MeshVolume represents the three-dimensional volume of the segmented carotid artery and therefore reflects overall vessel size.

$$Mesh \, Volume \, = \sum\limits_{i = 1}^{{N_{f} }} {\frac{{Oa_{i} \left( {Ob_{i} x \, Oc_{i} } \right)}}{6}}$$

The volume of the ROI *V* is calculated from the triangle mesh of the ROI. For each face *i* in the mesh, defined by points *a*_*i*_, *b*_*i*_ and *c*_*i*_, the (signed) volume *V*_*f*_ of the tetrahedron defined by that face and the origin of the image (*O*) is calculated. Mesh Volume is the total volume of ROI. This feature was useful in discriminating between SS and AS mice at three months; this could be interpreted as indicating that SS carotid arteries were slightly larger than AS carotid arteries, which was consistent with our previous findings [18, 19].

**Table 3:** Discriminant Radiomics Features obtained from radiomics analysis of three-month-old mouse cases

Discriminant Radiomics Features	*P*-value (< 0.05)
Original shape MeshVolume	0.0020

**Fig. 2:**
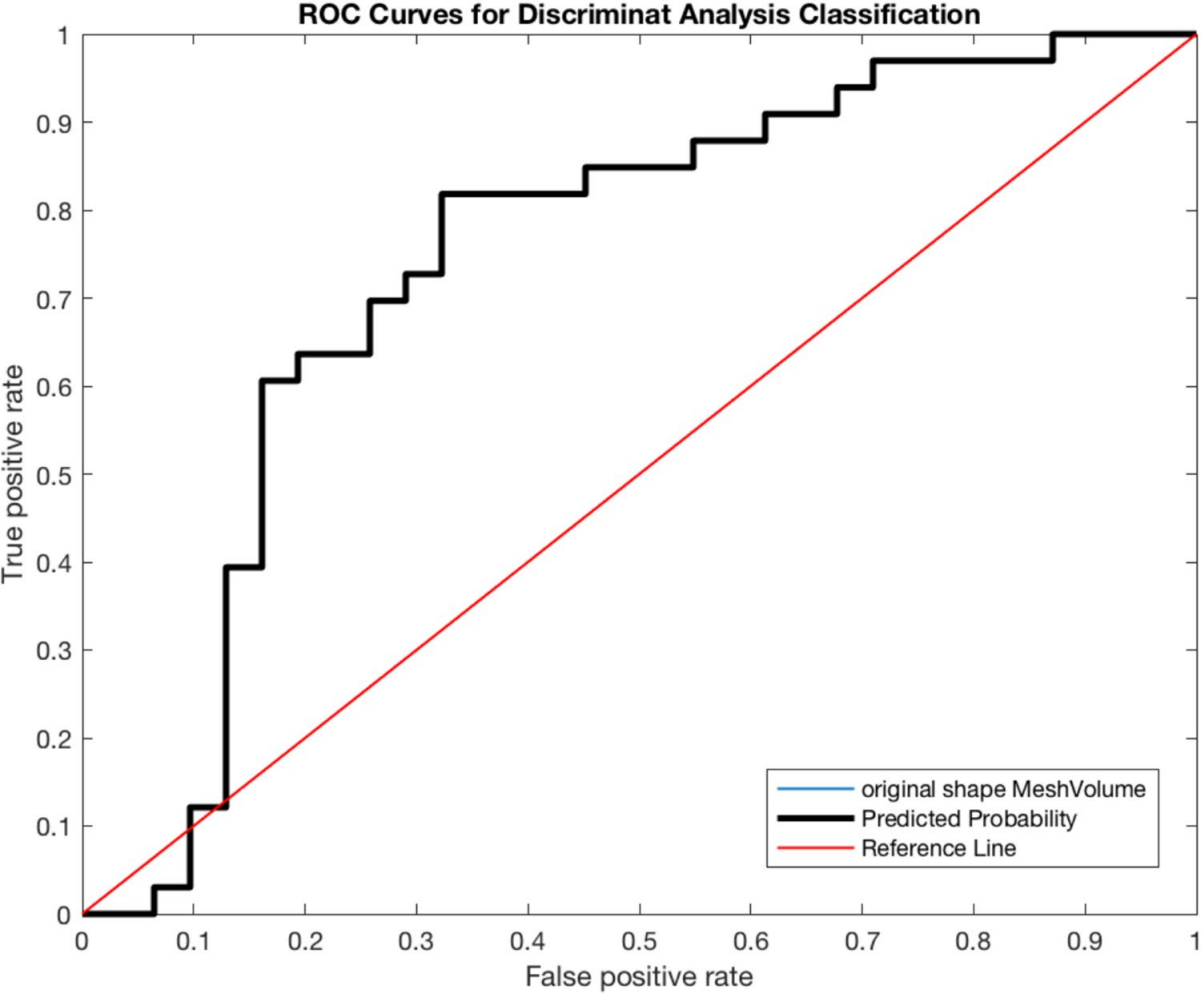
ROC curve relating to the radiomic feature analyzed (shape MeshVolume) in mice at 3 months of age. MeshVolume showed statistically significant discrimination between SS and AS, with an AUROC of 0.74; 95% CI between 0.61 and 0.87 with *p*-value of 0.0020

This feature presents high diagnostic performance in discrimination between SS and AS, with an AUROC of 0.67; 95% CI between 0.61 and 0.87 with p-value of 0.0020, sensitivity of 67.22%; 95% CI between 54.58 and 78.18, specificity of 83.88%; 95% CI between 72.58 and 91.86, an accuracy of 76.03%; 95% CI between 63.79 and 85.73 and a positive predictive value % (PPV) of 78.57%; 95% CI between 66.55 and 87.81. Figure 2 shows the ROC curve for the one most discriminated radiomics feature in mice at 3 months of age (Fig. 2).

### Investigation of Underlying Causes of Radiomics Misclassification

**Fig. 3:**
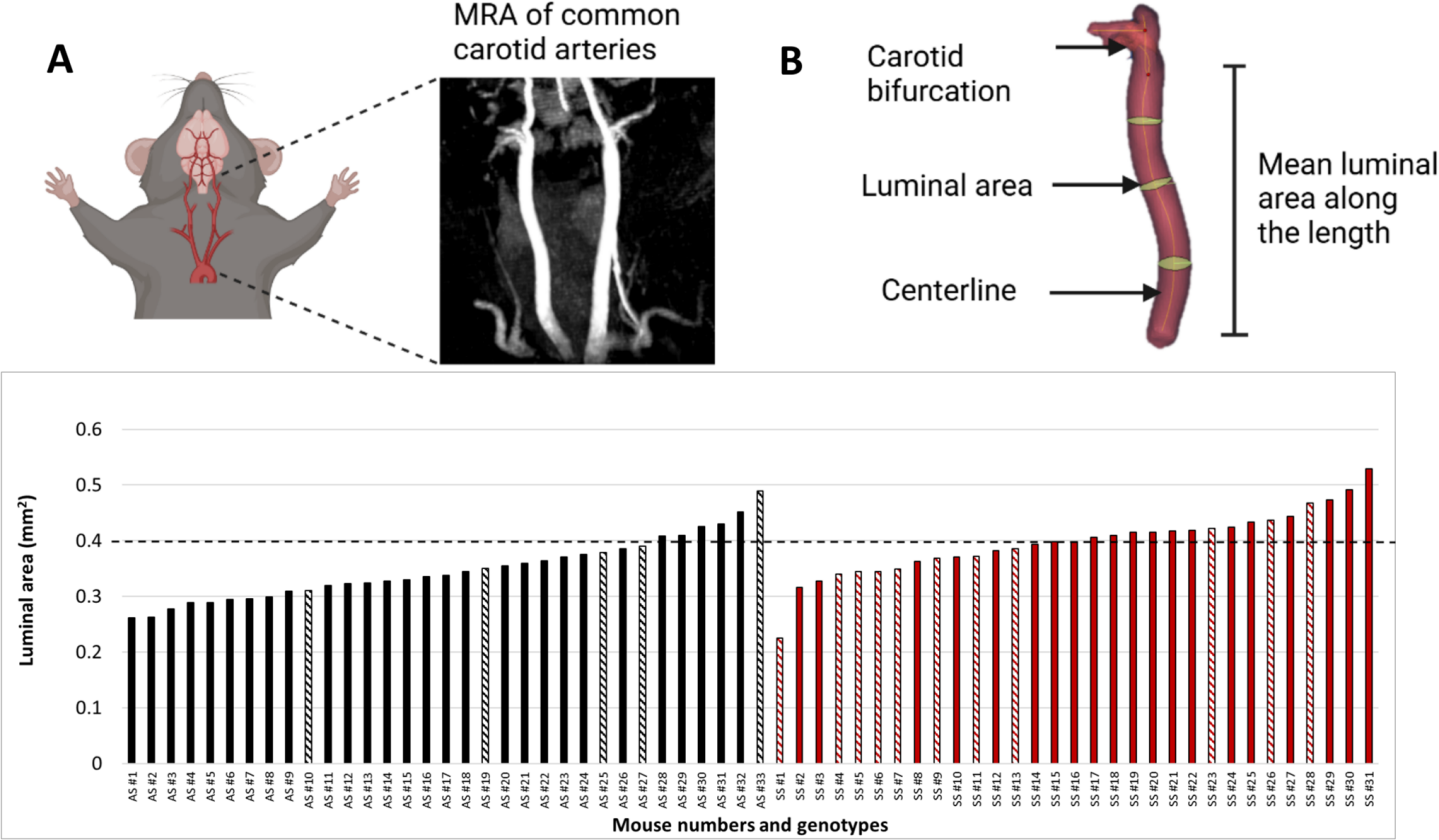
Mice that were misidentified by radiomics features are more likely to have artery luminal area measurements of the incorrect genotype. **a** SS and AS mice were scanned by MRA at 3-months of age. 3D reconstructed carotid artery images were segmented and reconstructed using *Mimics* software and morphological measurements of luminal cross-sectional area were extracted from the left side and averaged along the length to compare mouse-specific arterial growth and remodeling. The artery reconstruction was generated using *Mimics* and labels and figure enhancements was created using BioRender.com. **b** Carotid luminal areas of the common carotid arteries in AS (black) and SS (red) mice at 3 months of age, arranged in ascending order by genotype. Dashed bars indicate mouse cases that were incorrectly identified by the key radiomic feature. Of the incorrect mice, 4 were AS males, 1 AS female, 7 SS males, and 4 SS females. SS luminal artery areas are generally larger than AS, but those that were misclassified are generally smaller than other SS arteries

Radiomics feature extractions and analyses from MR scans do not assess anatomical and morphometric information directly, and thus are not direct inputs into radiomics analysis. However, we previously showed that morphometry of carotid arteries showed expansive remodeling and larger luminal cross-sectional areas in SS mice, measured first with histology of excised arteries [19] and subsequently through 3D reconstructions of MR-imaged arteries in living mice [28]. Using the expectation that SS mice had larger areas than AS mice, we next asked if mice incorrectly identified by radiomics features actually had measured luminal cross-sectional areas of the incorrect genotype or fell within the overlapping range of biological variability observed between groups. To make this comparison, all animal scans were lined up according to average luminal areas along the length of the common carotid artery to determine if there was a correlation among luminal areas and incorrect predictions. As expected, SS mice had luminal areas that are close to or above the 0.4 mm^2^ line due to SCD-mediated arterial remodeling while AS mice had smaller luminal areas (Fig. 3). The incorrect predictions using radiomics analysis are shown in dashed bars for each AS and SS mice. Of the incorrect mice, 4 were AS males, 1 AS female, 7 SS males, and 4 SS females. Three of the five AS mice that were incorrectly predicted had larger luminal areas and were closer to or above the 0.4 mm^2^ luminal area line, and 8 out of the 11 SS mice that were incorrectly predicted had smaller luminal areas and were below the 0.4 mm^2^ luminal area line (Fig. 3). This highlights that radiomic features capture gradual differences in arterial structure, consistent with expected biological variability in arterial remodeling in SCD. As a result, animals with overlapping structural characteristics across genotypes are expected to be more difficult to classify by this method.

### Prediction Results of Mice Having Both 1- and 3-Month Scans

Among the mice, 25 mice were scanned twice: at 1 month and again at 3 months of age. The next question was whether an incorrect radiomic features identification from a one-month-old scan would still be incorrectly identified for that same mouse at 3 months of age, or if 3 months of age would be more likely to be correctly identified than at 1 month of age. The rationale is that SS mice present more pronounced disease phenotypes as they age, and our hypothesis was that a more pronounced phenotype would be more distinguishable by radiomics features.

For the 25 mice that were scanned at both 1 month and 3 months of age, there was a distribution of correct and incorrect predictability shown in Table 4. Overall, 56% of the scans were correctly classified at both ages, while only 8% were misclassified at both 1 and 3 months. The remaining mice exhibited changes in classification status between ages, with comparable numbers transitioning from incorrect to correct (20%) and from correct to incorrect (16%) predictions, the majority of which were SS mice. These shifts likely reflect biological variability and changing arterial morphology as the mice age.

**Table 4:** Prediction accuracy for mice scanned at one month and three months of age

1-month prediction	3-month prediction	Number of cases (genotype)	Percentage (%)
Correct	Correct	14 (8 AS/6 SS)	56
Incorrect	Incorrect	2 (1 AS/1 SS)	8
Correct	Incorrect	4 (1 AS/3 SS)	16
Incorrect	Correct	5 (1 AS/4 SS)	20

**Fig. 4:**
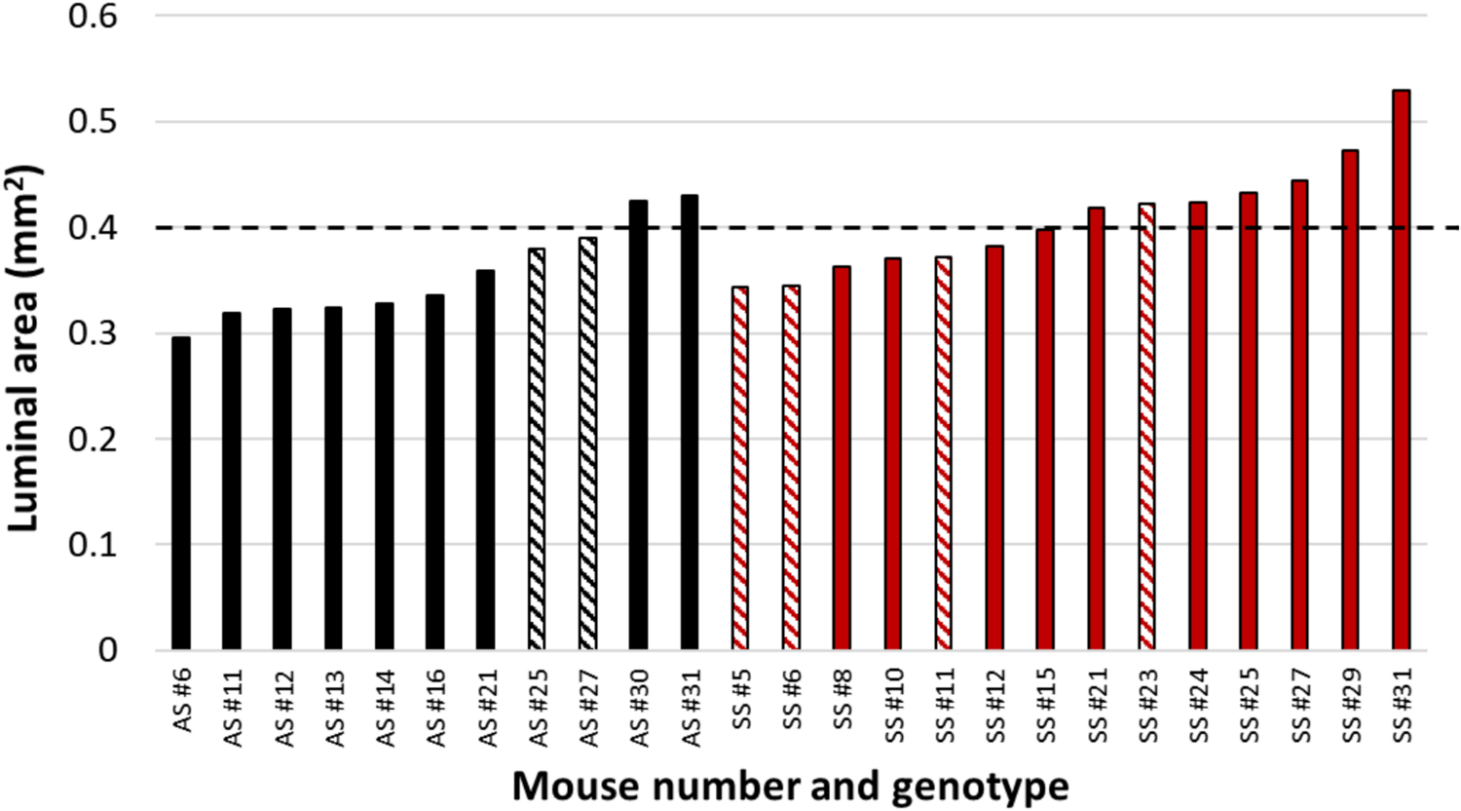
Artery luminal area measurements of mice scanned at two ages. SS and AS mice were scanned by MRA at one month and 3 months of age. Images were reconstructed and morphological measurements of luminal cross-sectional area were extracted as described above. Carotid luminal areas in AS (black) and SS (red) mice at 3 months of age, arranged in ascending order by genotype. Dashed bars indicate mouse cases that were incorrectly identified by the key radiomic feature. Of the incorrect mice: 1 AS male, 1 AS female, 1 SS male, and 3 SS females

Three-month luminal areas of the common carotid arteries were graphed for the 25 mice that were scanned at both 1 and 3 months (Fig. 4). Those in dashed bars were incorrectly identified at 3 months of age using radiomics features and discriminant analysis. Of the incorrect mice: 1 AS male, 1 AS female, 1 SS male, and 3 SS females.

Three out of the four incorrectly predicted SS mice had smaller luminal areas resembling those of AS mice, while both AS that were misclassified had higher luminal areas close to the 0.4 mm^2^ line.

### Image Fidelity or Arterial Structures that Confound Radiomics Feature Predictions

**Fig. 5:**
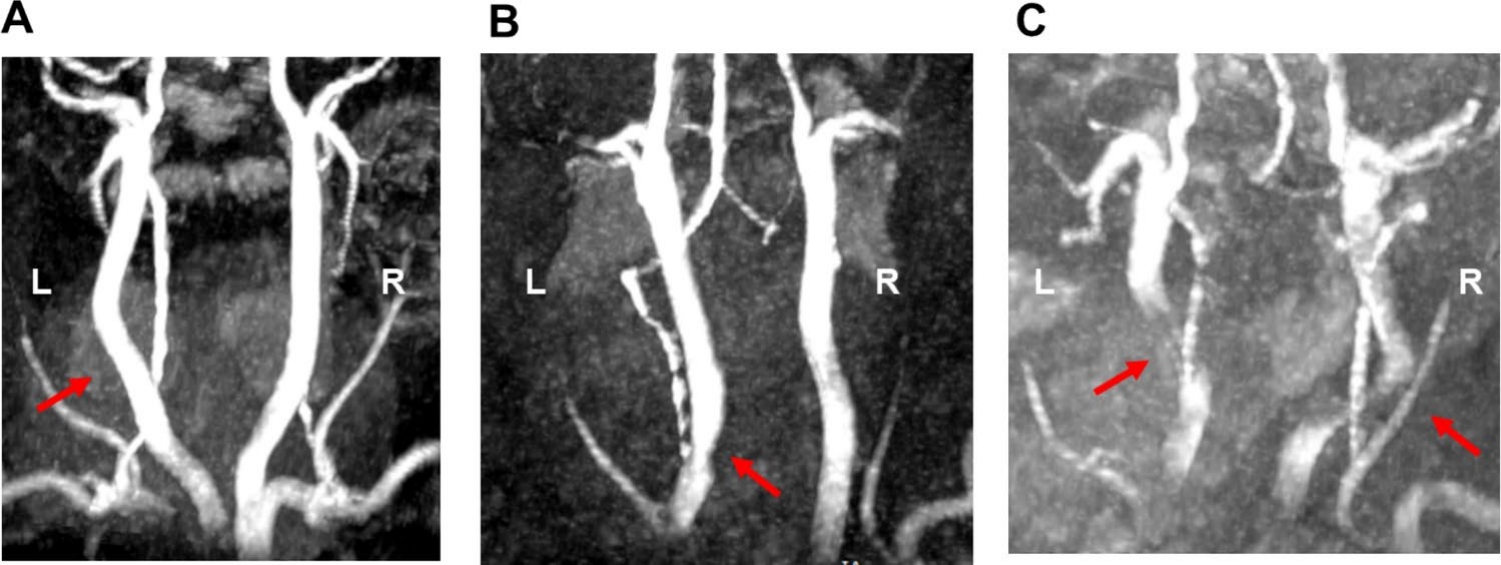
MRA scans from mice that were incorrectly predicted had abnormal features. **a** A 3-month-old AS mouse with increased curvature in the left common carotid artery was misidentified as SS. **b** A 3-month-old AS mouse with a region of reduced signal intensity and apparent luminal narrowing in the left common carotid artery was misidentified as SS. **c** A 3-month scan of an SS mouse with higher curvature in both common carotid arteries. The localized signal reduction observed on the MIP is consistent with curvature-related flow signal loss, a known limitation of TOF MRA. Additional technical factors, including RF power calibration variability, cannot be fully excluded as contributors to overall image appearance. Red arrows indicate the area of interest in the scan

Finally, to gain insight into reasons for radiomic feature misclassification, the MRA scans from mice that were incorrectly classified were examined to determine if artery abnormalities or errors in the MR scan were generating data that would drive the incorrect predictions from extracted radiomic features. Several abnormalities were observed in different mice including a 3-month-old AS mouse with increased curvature of the left common carotid artery not normally seen in AS mice (Fig. 5a). Another AS mouse had a region of reduced signal intensity and local luminal narrowing in the left common carotid not normally seen in AS mice (Fig. 5b, red arrow). In addition, one SS mouse had increased curvature of the common carotid arteries with localized signal reduction consistent with curvature-related flow signal loss on TOF MRA (Fig. 5c).

## Discussion

To our knowledge, this is the first study to demonstrate the feasibility of applying radiomics analysis to label-free carotid artery MRA to discriminate arterial phenotypes and predict genotype in a preclinical sickle cell disease model. Genotype discrimination was used as an initial feasibility study to assess whether radiomic features are sensitive to subtle arterial differences present in MRA with morphometric analyses of cross-sectional areas performed separately to aid interpretation. No histological validation was performed, and therefore these results should not be interpreted as predictors of stroke risk, but could be indicators of arteriopathy. Instead, the identified distinct radiomic features highlight the potential of non-invasive imaging to capture arterial physiological changes associated with SCD at different disease stages and each indicate where the heterozygous condition may have crossover susceptibility to arterial damage. MRA imaging, along with radiomics and advanced machine learning techniques, offer a non-invasive approach to assess vascular changes in preclinical models of SCD. Radiomics can contribute to the broader understanding of how SCD affects vascular biology, particularly in large arteries like the common carotid arteries, and even how the range of severity for sickle cell trait affects arteries. This work provides a methodological foundation for future studies linking radiomic features to arteriopathy, hemodynamics, and disease severity. Further research is warranted to determine how these imaging-derived features relate to clinically relevant arterial changes in SCD.

In mice at one month of age, a combination of four radiomic features achieved an accuracy of 74%, suggesting that these features capture early vascular changes in SCD. In contrast, at three months of age, a single feature, mesh volume, provided a slightly higher prediction performance (accuracy of 76%), despite increased variability in disease severity. While the AUROC achieved in our study demonstrates good performance, there is room for improvement. Not all mice with SCD experienced arterial expansive remodeling, and the majority 8 out of 11 of the incorrectly predicted SS mice had smaller luminal areas compared to other SS mice at 3 months of age (Fig. 3). Humans with AS genotypes, referred to as sickle cell trait, have no increased risk for strokes [29]. AS mice imitate sickle cell trait in humans [30]. Some AS mice had arterial expansive remodeling signs including enlarged luminal areas of their common carotid arteries and increased curvature, resembling features observed in mice with SCD. This finding suggests that the AS genotype may not be entirely benign in arterial damage. In addition, there were some cases that were incorrectly identified that could indicate discrepancies between the predicted features and the actual biological conditions. For example, abnormalities such as luminal narrowing and increased curvature were evident in some MRA scans, contributing to the discrepancy in quantitative predictions. In addition to luminal size, arterial curvature represents a quantifiable geometric feature that may reflect arterial remodeling in SCD. Future work could assess whether SS mice exhibit increased common carotid artery curvature and altered branching patterns relative to controls, and whether similar features are present in SCD patients. Structural changes in the common carotid artery may also be accompanied by compensatory remodeling elsewhere in the vascular tree, such as altered branching or arteriogenesis. Established methods exist to quantify geometric curvature in murine vessels using imaging-derived centerlines [31], highlighting approaches that could be extended to carotid curvature analysis in SCD models.

This study has several limitations beyond those described above. Body temperature was not monitored during imaging. Although continuous body heating with a circulating warm water heating blanket was used, subtle temperature-related effects on vessel caliber cannot be fully excluded. Luminal artery size is influenced by factors such as sex and body weight, which were not modeled in this analysis and may contribute to subject-to-subject variability. We performed analysis of male and female data combined due to the small dataset, but showed data disaggregated by sex with the predictability. However, future studies with larger cohorts will require disaggregating the data to determine any potential sex-specific differences as male individuals with SCD generally have higher mortality rates and are more prone to complications compared to females [32]. In addition, although a subset of mice was imaged longitudinally at both ages, the analyses treated time points independently. Future longitudinal studies with larger datasets will benefit from paired statistical tests and mixed-effects modeling approaches to account for within-subject correlations and better characterize age-dependent radiomic changes. Neck positioning during prone imaging may influence apparent carotid artery curvature on TOF MRA, but this was avoided by using consistent positioning across animals to minimize these effects. Assessment of arterial curvature and evaluation in older animals represents an important direction for future work. Furthermore, cases showing apparent luminal narrowing on MRA may reflect flow-related signal loss due to complex or curved flow, a known limitation of TOF MRA. Confirmation of true stenosis would require flow-perpendicular 2D imaging or contrast-enhanced MRA, which were beyond the scope of this study. However, in other studies where we have used ultrasound and micro-CT on carotid and cerebral arteries from SS and AS mice, constrictions and expansions were identified along the lengths of the arteries [33], which suggests that not all of the perceived stenoses in the MRAs are just artifacts of the TOF MRA. Our findings are specific to the Townes mouse model of SCD and may not directly translate to humans with SCD without validation in clinical settings. External validation across scanners and sites will be important to determine the robustness and generalizability of these radiomic features. Future studies will be needed to evaluate whether similar imaging-derived radiomic features are observed across other preclinical SCD models (e.g., Berkeley) and across human genotype variability (e.g., HbSC).

Mounting evidence suggests that SCD-mediated vascular changes are multifactorial, involving inflammatory signaling pathways, proteolytic enzymes, and remodeling of arterial walls [19]. Interventions such as bone marrow transplant (BMT) [18] and hydroxyurea initiation [34] can alleviate or delay vascular damage, but the clinical decision-making around these therapies still relies on relatively limited biomarkers, including TCD velocities and broader clinical parameters. As observed in other vascular conditions like coronary heart disease or diabetic vasculopathy, adding radiomics-based imaging metrics to standard clinical models improves diagnostic and prognostic accuracy [13, 35]. Translating these methods to SCD may similarly refine our ability to classify arterial remodeling severity, predict stroke risk, and monitor treatment response.

Taken together, these findings highlight an urgent need to integrate radiomics analysis with non-invasive MRI/MRA for SCD-related arteriopathy. The potential for this technique to detect subtle textural or volumetric features of arterial remodeling could revolutionize how clinicians screen for, diagnose, and treat high-risk individuals with SCD, ultimately reducing stroke incidence and related complications [34, 36].

## Data Availability

Corresponding authors will make data available to inquiries upon request.

## Abbreviations

SCD: Sickle cell disease
HbS: Sickle hemoglobin (HbS)
MRI/MRA: Magnetic resonance imaging and angiography
AUROC: Area under the receiver operating characteristic curve
TCD: Transcranial Doppler ultrasound
